# The Impact of a Single Hip Manipulation on Quadriceps Activity and Performance: A Randomized Study

**DOI:** 10.3390/biomedicines13040900

**Published:** 2025-04-08

**Authors:** Rafał Studnicki, Monika Sochaj, Karol Skup, Bartłomiej Niespodziński, Piotr Aschenbrenner, Radosław Laskowski, Piotr Łuczkiewicz

**Affiliations:** 1 Department of Physiotherapy, Medical University of Gdańsk, 7 Dębinki Street, 80-211 Gdańsk, Poland; 2 Student Scientific Circle of Orthopaedic Physiotherapy, 2nd Division of Orthopaedics & Kinetic Organ Traumatology, Medical University of Gdańsk, 80-211 Gdansk, Poland; monikasochaj@gumed.edu.pl (M.S.); skupkarol@gumed.edu.pl (K.S.); 3 Department of Biological Foundations of Physical Education, Faculty of Health Sciences and Physical Education, Kazimierz Wielki University, Sportowa 2, 85-091 Bydgoszcz, Poland; bar.niespodzinski@wp.pl; 4 Department of Physical Education, Gdansk University of Physical Education and Sport, 80-211 Gdansk, Poland; piotr.aschenbrenner@awf.gda.pl; 5 Department of Physiology, Gdansk University of Physical Education and Sport, 80-211 Gdansk, Poland; radoslaw.laskowski@awf.gda.pl; 6 2nd Division of Orthopaedics & Kinetic Organ Traumatology, Medical University of Gdańsk, 80-211 Gdansk, Poland; piotr.luczkiewicz@gumed.edu.pl

**Keywords:** musculoskeletal manipulations, electromyography, muscle strength, knee joint, hip joint

## Abstract

**Background/Objectives**: Optimal activation of the quadriceps femoris, particularly the vastus medialis, while minimizing excessive activation of the vastus lateralis, is crucial for treating knee injuries like ACL ruptures and patellofemoral pain syndrome. Restoring proper muscle balance may enhance rehabilitation outcomes, but effective strategies for modulating muscle activity remain unclear. High-velocity low-amplitude hip manipulation has shown potential to influence neuromuscular function, yet its impact on quadriceps activation during knee extension has not been well studied. Therefore, the main aim of this study is to examine the effects of a single session of high-velocity low-amplitude hip manipulation on quadriceps femoris muscle activation and maximum voluntary contraction during knee extension. **Methods**: This study utilizes a randomized controlled design. Thirty physically active men and women (mean age: 21.9 ± 1.7 years) were randomly assigned to either an experimental group (n = 15; receiving hip joint manipulation) or a control group (n = 15; undergoing a sham intervention). Participants in the intervention group received a treatment involving hip manipulation and short-duration traction. Muscle activity of the rectus femoris, vastus lateralis, and vastus medialis was assessed using surface electromyography before and after the intervention, while muscle performance was measured by evaluating isometric knee extension strength in the lower limb. The isometric strength test was conducted in a seated position with the knee flexed at 60 degrees in Biodex System 4. **Results**: This study finds that the experimental group had significantly higher vastus lateralis mean amplitude (*p* = 0.020; effect size = 0.186) and vastus medialis mean amplitude (*p* < 0.001; effect size = 0.577) of electromyography root mean square electromyography compared to the control group. The experimental group also showed greater vastus medialis max amplitude (*p* < 0.001; effect size = 0.435). No significant differences were noted for rectus femoris mean amplitude (*p* = 0.078; effect size = 0.110), vastus lateralis max amplitude (*p* = 0.363; effect size = 0.031), rectus femoris max amplitude (*p* = 0.069; effect size = 0.117), or median frequency of the raw electromyography signal across muscle groups. **Conclusions**: In conclusion, high-velocity low-amplitude hip manipulation significantly enhances vastus medialis activation, highlighting its potential to improve quadriceps balance. These findings support the incorporation of hip manipulation into rehabilitation protocols.

## 1. Introduction

Knee injuries, affecting both young individuals and adults, are a prevalent issue in musculoskeletal health [1,2]. For example, reduced quadriceps strength was associated with an increased risk of further cartilage deterioration in the lateral patellofemoral joint in women. This implies that improving quadriceps strength could potentially help prevent the progression of structural damage in the patellofemoral joint in women [3]. One of the key factors driving this degeneration is the weakening of the quadriceps femoris (QF) muscle, a critical muscle group that plays a central role in maintaining knee stability and proper biomechanics [4].

The quadriceps muscle is essential for stabilizing the knee joint during movement, and any deficit in its strength or neuromuscular control can significantly alter knee biomechanics, leading to instability [5,6]. Neuromuscular impairments, such as the inhibition or abnormal activation of the quadriceps, can result in clinical symptoms that disrupt normal joint function, increasing the risk of further injury [7]. Proper neuromuscular control, therefore, is essential for achieving active joint stability, which supports safe and efficient movement in daily activities or during training [8].

There is growing evidence suggesting that addressing hip joint biomechanics, particularly in relation to hip abduction, external rotation, and neuromuscular coordination, is crucial for mitigating knee dysfunction [9]. A specific focus on the role of the hip external rotators, such as the gluteus maximus, piriformis, and deep hip rotators, may help in improving lower limb alignment and function [10]. Dysfunction or weakness in these external rotators can contribute to poor control of the femur during dynamic movements, increasing the risk of knee valgus and altering lower limb kinematics [11]. For example, knee valgus has been linked to decreased strength in the hip extensor muscles [12]. Additionally, an increased Q-angle (the angle formed between the quadriceps muscle and the patellar tendon) has been implicated in altered patellar tracking, which may compound issues related to knee valgus [13]. This altered Q-angle, often resulting from dysfunctional hip mechanics, may place additional strain on the knee joint, contributing to conditions such as patellofemoral pain syndrome and anterior cruciate ligament injuries [14,15,16,17].

Physiotherapists have increasingly focused on strengthening the QF muscle to restore knee function [18]. Open and closed kinetic chain exercises have been widely used in rehabilitation programs to enhance quadriceps strength, while also targeting the hip adductors and external rotators to improve overall lower limb function and alignment [19,20,21]. Clinical research has shown that quadriceps training can significantly reduce knee pain, with a systematic review providing strong evidence for its efficacy [22]. Additionally, studies such as the one by Fukuda et al. [23] suggest that combining quadriceps strengthening with exercises targeting the hip abductors and external rotators yields better results in terms of pain relief and knee function compared to quadriceps training alone.

Recent research has highlighted the potential of manual therapy to enhance neuromuscular function [24]. Among various techniques, the high-velocity low-amplitude (HVLA) technique is one of the oldest and most commonly used in chiropractic care. It encompasses different forms, including the diversified technique, Gonstead adjustment, and Thompson Terminal Point technique [25]. Manipulation, typically characterized by high-velocity low-amplitude thrusts, is commonly used in cases of joint pain or restricted range of motion [26]. Its effects extend beyond biomechanical changes, also engaging neurophysiological and supraspinal mechanisms that impact muscle function and neuromuscular control [27]. For example, an HVLA hip distraction has been shown to enhance gluteus maximus strength in individuals with knee injuries and weakness in the lower extremities [28]. In another study [29], manipulations involving high-velocity low-amplitude in the ankle area may help boost hip abductor strength in people with a history of ankle sprains and unilateral weakness, as assessed during a tensor fascia latae muscle test.

Despite the growing body of research on manipulation [30,31], the immediate effect of lateral hip manipulation on quadriceps muscle function has not been thoroughly explored. Given the interconnectedness of hip and knee biomechanics [32], investigating this relationship could provide valuable insights into novel rehabilitation strategies for individuals with quadriceps weakness, patellofemoral pain, or knee degeneration. This study aimed to assess the immediate effects of a single lateral hip manipulation on quadriceps muscle activation, measured using electromyography (EMG), and neuromuscular function, measured using isometric knee extension strength.

## 2. Materials and Methods

This research adhered to the Consolidated Standards of Reporting Trials (CONSORT) guidelines for documenting randomized experimental studies [33].

### 2.1. Trial Design

To assess the impact of hip manipulation on quadriceps femoris neuromuscular performance, a prospective, randomized double-blind design was implemented. Participants were randomly assigned to two equal groups: one group underwent the intervention (manipulation), while the other group received a sham treatment. The allocation ratio for both groups was 1:1. A 1:1 allocation ratio means that each participant has an equal probability of being assigned to either the intervention or sham treatment group. This ensures that the groups are balanced in size, reducing selection bias and improving the validity of comparisons between them.

Approval for the study was granted by the Independent Bioethics Committee for Scientific Research at the Medical University of Gdańsk (Resolution No. NKBBN/866/2022-2023). Participants received a comprehensive explanation of the study protocol in clear, accessible language, and written informed consent was secured from each volunteer. This consent explicitly indicated that participants could withdraw from the study at any point without facing any penalties. The research adhered to the ethical principles for studies involving human subjects as outlined in the Declaration of Helsinki.

### 2.2. Participants

This research was carried out following the specific inclusion criteria established beforehand: (i) participants must be healthy individuals (ii) aged between 19 and 26 years and (iii) able to attend all intervention and assessment sessions. The exclusion criteria included (i) any history of surgery involving the lower limbs or lumbar spine, (ii) recent lower limb injuries occurring within the past 6 months, (iii) experiencing pain in the ankle, knee, or hip, (iv) ankle joint hypermobility, (v) presence of rheumatic diseases, (vi) neurological disorders, (vii) cancer-related conditions, (viii) connective tissue disorders, (ix) symptoms indicative of spinal root compression, sciatica, or spinal canal stenosis, and (x) prior treatment involving manipulation.

**Figure 1:**
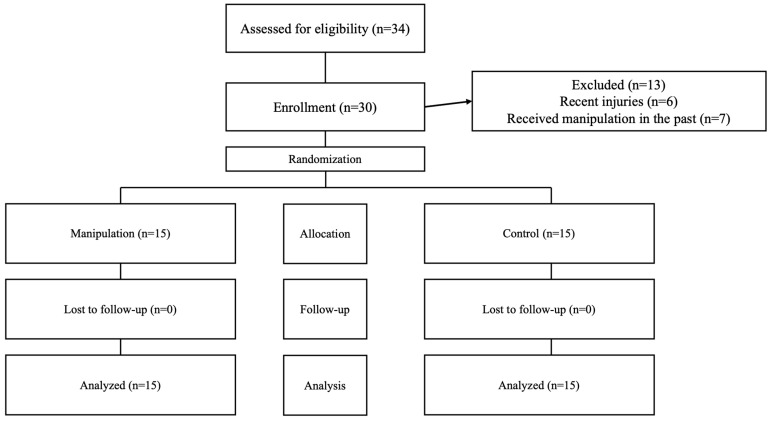
Flowchart showing participant progression throughout the study steps.

Participants were recruited through announcements in the primary intervention area, social media outreach, and direct contact. This straightforward sampling approach initially attracted 43 volunteers. After screening, 13 were excluded, resulting in a final group of 30 eligible individuals, both men and women, who expressed a desire to participate. Among these, 22 were men and 8 were women, all of whom met the established criteria for group assignment (see Figure 1).

### 2.3. Interventions

Participants in the intervention group received a treatment HVLA manipulation and short-duration traction [34]. The procedure was conducted as follows: Participants were first informed about the intervention’s nature. They were positioned supine with a 30° flexion at the hip joint and their upper limbs resting alongside their trunk. The therapist, an expert with over 25 years of experience, positioned themselves laterally to the participant, carefully wrapping both hands around the proximal thigh, as close to the pelvis as possible. Then, the therapist performed a thigh movement, moving it laterally, parallel to the ground, until the maximum felt resistance of the tissue tension in the hip joint was reached. From this point, a distance of 1 cm was measured between the outer side of the patient’s thigh and the therapist’s body to standardize the repeatability of subsequent interventions. At this point, the therapist made sure that the participant did not feel any discomfort and then performed a 1 s lateral manipulation. This technique was performed once for each participant.

Participants in the control group received a sham intervention. This procedure closely mirrored the HVLA manipulation, but during the lateral movement of the thigh, no actual tissue tension was applied. When the thigh reached the lateral position, the therapist used their torso to prevent any further lateral movement, thereby inhibiting any increase in tissue tension at the hip joint. The therapist confirmed that the participant did not experience discomfort, and then executed a movement that mimicked the manipulation without applying any additional tissue tension [16]. The temperature of the room was kept at 22 °C, with a relative humidity level of 50%.

### 2.4. Outcomes

Located at the Academy of Physical Education in Gdańsk, the Physical Exercise Laboratory of the Department of Biomechanics and Sports Engineering served as the study venue. To enhance the reliability of the therapeutic assessment, each volunteer was tested twice by the same physiotherapists. The measurement team consisted of three physiotherapists, each holding a PhD degree and with a minimum of 5 years of experience in assessing muscular activity using EMG and in measuring muscular strength with the Biodex system. Measurements were taken both before and immediately after the manipulation or placebo of the hip joint.

To ensure repeatability and objectivity, the Biodex System 4 dynamometer (Biodex Medical Systems, Inc., Shirley, NY, USA) chair settings were utilized for better patient stabilization, following the manufacturer’s guidelines. During the initial assessment, the settings for the movable components (Positioning Chair) were recorded and replicated for the second assessment. Participants were secured using leather straps. In this position, each participant performed three maximum voluntary isometric contractions (MVIC) lasting 5 s each. A 30 s rest was provided between each repetition, and participants received verbal encouragement to maximize their effort during the measurements.

#### 2.4.1. Surface Electromyography

Surface electromyography (SEMG) data were recorded from the quadriceps femoris (QF) during the MVIC of the knee extension. Specifically, the signals from the rectus femoris (RF), vastus lateralis (VL), and vastus medialis (VM) muscles were collected. Electrode placement and skin preparation—including shaving, abrading, and cleansing with alcohol—were conducted in accordance with the SENIAM guidelines.

During each 5 s MVIC, the middle 3 s of data were analyzed. The SEMG signals were differentially amplified with a gain of 500 using the TeleMyo DTS system (Noraxon, Scottsdale, AZ, USA) and Ag/AgCl 1-cm^2^ surface electrodes (Sorimex, Toruń, Poland). The signals underwent band-pass filtering between 15 and 500 Hz and were sampled at 1500 Hz with 16-bit resolution via an analog-to-digital converter. Subsequently, the SEMG data were archived and processed using MyoResearch 2.8 software (Noraxon, Scottsdale, AZ, USA) [30].

Signal processing included full rectification and smoothing via the root mean square (RMS) method, utilizing a 100 ms moving time window. Average values from all three MVIC trials were calculated, including the mean and maximum amplitude of electromyography root mean square (EMG_RMS_, measured in microvolts) and the median frequency of the raw SEMG signal power spectrum (EMG_MED_, in Hz). Additionally, percentage changes from pre-manipulation to post-manipulation measurements were computed for analysis. Finally, the VL-VM ratio, calculated by dividing the EMG values of VL by those of VM, was also determined pre- and post-intervention.

#### 2.4.2. Isometric Knee Extension Strength

Using the Biodex System 4 (Biodex Medical Systems Inc., Shirley, NY, USA), the muscle strength of the knee extensors was evaluated. According to the manufacturer’s guidelines, participants were seated in the device with their back supported, their tested lower limb fully extended at the knee joint (neutral in both sagittal and frontal planes), and the hip positioned at 90° flexion. The lower limb was secured to the device’s arm with leather straps, ensuring that the device’s rotation shaft aligned with the knee joint’s anatomical axis for movement in the sagittal plane. Each participant then performed three MVIC, each lasting 5 s, with a 30 s rest between repetitions [35]. Participants received verbal encouragement to maximize their effort during each contraction. For further analysis, the highest peak torque (Nm) from the three attempts was chosen, and peak torque was normalized to each participant’s body mass (Nm × kg^−1^).

### 2.5. Sample Size

Using G*Power software (version 3.1.9.6, Universität Düsseldorf, Düsseldorf, Germany), the sample size for the study was calculated. The analysis was based on an ANCOVA model, which assumed a moderate effect size of 0.6 and a power of 0.85 for two groups [34]. This approach resulted in a suggested sample size of 28 participants.

### 2.6. Randomization

Participants were assigned identification numbers in a 1:1 ratio through a simple randomization process using Research Randomizer software (Social Psychology Network, Wesleyan University, Middletown, CT, USA). To maintain allocation concealment, participants were randomized and assigned prior to the initial evaluation. There were no alterations to group assignments for any of the participants.

### 2.7. Blinding

Participants were kept unaware of the intervention, preventing them from observing others, while a control therapy was administered to the control group to ensure uniform conditions across all groups. Additionally, the evaluators conducting the assessments were blinded to the group assignments of the participants.

### 2.8. Statistical Methods

Prior to conducting any inferential analyses, the normality of the sample was assessed using the Shapiro–Wilk test. Although some variables did not show normality (*p* < 0.05), the sample size of 30 is sufficient for the Central Limit Theorem to apply [36], which allows us to assume that the sampling distribution of the mean is approximately normal. As such, these variables are considered normal for the purposes of parametric analysis. Additionally, the homogeneity of variance assumption was examined using Levene’s test, with results indicating no significant violation (*p* > 0.05). Based on these findings, no violations of the assumptions for parametric tests were observed, supporting the use of parametric inferential methods for the analysis.

An ANCOVA was conducted to compare the intervention and control groups, using baseline levels as a covariate. This analysis aimed to evaluate the differences in post-intervention outcomes between the two groups while controlling for initial variations. The effect size was calculated using the partial eta squared ($\eta_{p}^{2}$), and the magnitude of the differences was interpreted as follows [37]: 0.00–0.01, trivial; 0.01–0.06, small; 0.06–0.14, medium; >0.14, large.

The within-group differences were assessed using Cohen’s d, with the following thresholds for effect sizes [38]: 0.0–0.2 indicating a trivial effect, 0.2–0.6 representing a small effect, 0.6–1.2 denoting a moderate effect, and values greater than 1.2 signifying a large effect. Additionally, a repeated measures ANOVA was conducted to compare the percentage difference between post- and pre-intervention scores, calculated using the formula (post − pre)/pre × 100. Using IBM SPSS Statistics software (Version 29.0.2.0, Armonk, NY, USA: IBM Corp), statistical analyses were performed with a significance level set at *p* < 0.05.

## 3. Results

Of the 43 volunteers initially recruited, 6 were excluded due to lower limb injuries sustained in the previous six months, and 7 were excluded due to having received prior manipulation. After these exclusions, the remaining 30 participants were assigned to both groups and completed the intervention (Figure 1). All 30 participants were analyzed and included in the statistical evaluation.

Among the participants, 22 were men and 8 were women. The overall mean age was 22.0 ± 1.7 years, with an average height of 179.9 ± 9.5 cm and body mass of 75.5 ± 13.9 kg. A detailed breakdown of the main anthropometric and demographic data for each group is provided in Table 1.

Table 2 presents the descriptive statistics and inferential statistics comparing within-group differences (post–pre) for the main outcomes. The ANCOVA, using the baseline as covariable, revealed that experimental group presented significantly greater VL mean amplitude of EMG_RMS_ (*F* = 6.155; *p* = 0.020; $\eta_{p}^{2}$ = 0.186) and VM mean amplitude of EMG_RMS_ (*F* = 36.784; *p* < 0.001; $\eta_{p}^{2}$ = 0.577) than control, while no significant differences between groups were found on the RF mean amplitude of EMG_RMS_ (*F* = 3.349; *p* = 0.078; $\eta_{p}^{2}$ = 0.110).

**Figure 2:**
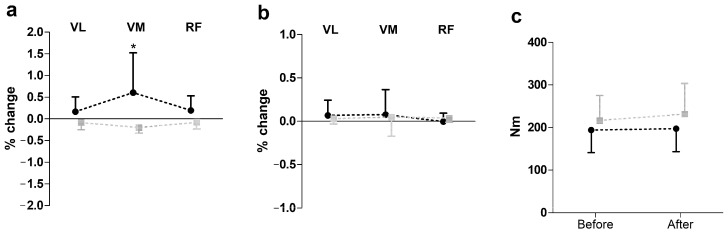
Impact of hip manipulation on surface electromyography (SEMG) and performance during knee extension. Black circles: hip manipulated group; gray squares: sham controlled group; VL: vastus lateralis; VM: vastus medialis; RF: rectus femoris. Percentage changes in surface electromyography amplitude (**a**), median frequency (**b**), and peak torque (**c**) * Significant difference with all other muscle in both groups except RF in hip-manipulated group at *p* ≤ 0.05.

Figure 2 presents the results of SEMG and peak torque changes (post-pre) following hip manipulation during knee extension. A two-way repeated measures ANOVA revealed a significant group effect on SEMG amplitude (F = 16.68, *p* ≤ 0.01, η^2^ = 0.37), with the hip manipulation group showing an increase in amplitude, while the control group showed a decrease. However, no significant effects were observed for peak torque during knee extension.

Table 3 presents the VL-VM ratio data. A significantly greater VL-VM ratio for the mean amplitude of EMG_RMS_ (F = 4.537; *p* = 0.042; $\eta_{p}^{2}$ = 0.144) was observed in the control group after the intervention. However, no significant differences between groups were found for the VL-VM ratio in the median frequency of the raw SEMG signal power spectrum (F = 0.023; *p* = 0.882; $\eta_{p}^{2}$ = 0.001) or for the maximum amplitude of EMG_RMS_ (F = 0.797; *p* = 0.380; $\eta_{p}^{2}$ = 0.029).

## 4. Discussion

Our study found that high-velocity low-amplitude hip manipulation resulted in significantly greater muscle activation in the VL mean amplitude of EMG_RMS_, as well as in the VM mean amplitude and maximum amplitude of EMG_RMS_, compared to the control group that only received a sham intervention. However, the intervention did not have a significant effect on the RF when compared to the sham intervention.

The VM benefited the most from the high-velocity low-amplitude hip intervention, showing significantly greater mean and maximum amplitudes of EMG_RMS_. Additionally, there were significant within-group enhancements in both variables, in addition to the previously mentioned between-group differences. Previous research [39] has indicated that similar manipulative techniques can effectively alter motor unit recruitment patterns in other muscle groups. For instance, studies on spinal manipulation have shown an increase in the recruitment of lower-threshold lower-twitch torque motor units, highlighting the broader implications of these interventions on muscular function. Additionally, a previous study [30] utilizing manipulation found it to be significantly effective in enhancing muscle activation in the infraspinatus compared to the control group. Finally, a study on hip manipulation [34] found a significant increase (*p* > 0.05) in EMG_RMS_ amplitude in the gluteus medius muscle.

High-velocity manipulations may stimulate the muscle’s neuromuscular pathways effectively, enhancing the recruitment of motor units and improving the firing rate [40]. The low-amplitude component could promote greater muscle activation while minimizing fatigue, allowing for sustained engagement of the VM. Moreover, such manipulations might facilitate increased blood flow and metabolic processes in the muscle, further contributing to enhanced muscle function [41]. The significant within-group enhancements suggest that the intervention not only produced immediate benefits, but also fostered ongoing improvements in muscle coordination and activation over time.

The observed enhancements in mean and maximum EMG_RMS_ in the VL and VM following hip manipulation may be explained by the activation and recruitment of motor units specific to these muscles. Both the VL and VM play essential roles in knee extension and stabilization, and the hip manipulation likely created an optimal biomechanical environment that facilitated improved neuromuscular communication and muscle engagement during the intervention [41]. Increased EMG_RMS_ values suggest that the manipulation enhanced the muscle’s ability to generate force effectively, possibly through increased motor unit synchronization and recruitment, which are known to influence muscle performance. Conversely, the lack of significant changes in the median frequency of the raw SEMG signal power spectrum may indicate that, while the overall muscle activation intensity increased, there were no substantial shifts in the muscle fiber recruitment pattern or the spectral properties of the electrical activity. This could be attributed to the fact that the manipulation primarily affected the magnitude of activation without altering the frequency, which may require more intensive or prolonged interventions to influence [42].

The pronounced effect of manipulation on the VM and VL, as opposed to the RF, can be attributed to the critical role within the quadriceps muscle group. For instance, VM is essential for stabilizing the patella and facilitating knee extension, particularly during activities that require dynamic knee stabilization [43]. By manipulating the hip, a more favorable mechanical environment may be established, enhancing the VM’s activation and function. In contrast, the RF did not exhibit significant differences between the intervention and control groups. This disparity could be due to the RF’s more complex role, as it also acts as a hip flexor in addition to its knee extension function [44]. The manipulation may not have sufficiently targeted the RF’s activation patterns, limiting its responsiveness to the intervention. This highlights the importance of understanding the specific biomechanical roles of each muscle in the quadriceps group when evaluating the effects of physiotherapeutic interventions.

Despite the findings of our study, there are several limitations that should be acknowledged. The sample size did not include sports athletes, which may limit the generalizability of the results to athletic populations. Additionally, the age group targeted in this study restricts the applicability of the findings to older populations, suggesting that future research should aim for a more diverse participant pool. This study’s focus on acute outcomes also leaves the long-term effects of HVLA hip manipulation on muscle activation uncertain; therefore, future studies should explore the chronic effects of such interventions over extended periods. Moreover, investigating variations in manipulation techniques and their specific impacts on different muscle groups—especially those, like the RF, that did not show significant changes—could offer deeper insights into neuromuscular dynamics. The use of advanced imaging or biomechanical assessments could also help elucidate the underlying mechanisms of action, particularly regarding motor unit recruitment patterns and the influence of muscle architecture on activation responses. A further limitation is the possibility that participants were able to distinguish between the real and sham interventions, despite efforts to carefully design the sham procedure to mimic the HVLA manipulation without applying tissue tension. The therapist’s skill and adherence to the protocol aimed to minimize biases; however, future studies could incorporate additional measures, such as blinding participants to the type of treatment, to reduce this risk further.

Despite the limitations, the findings of our study have implications for practitioners in the fields of physical therapy. The effectiveness of high-velocity low-amplitude hip manipulation in enhancing muscle activation, particularly in the VM and VL, suggests that incorporating this technique into rehabilitation protocols or warm-up could improve outcomes for participants recovering from knee injuries or those seeking to enhance athletic performance. Practitioners should consider the specific roles of these muscles in knee stabilization and extension when designing individualized intervention plans.

## 5. Conclusions

In conclusion, our study demonstrates that a single session of high-velocity low-amplitude hip manipulation enhances the activation of both the vastus lateralis (VL) and vastus medialis (VM) during knee extension, while having no significant effect on the rectus femoris (RF) when compared to a sham intervention. These findings suggest that high-velocity low-amplitude hip manipulation can effectively modulate quadriceps muscle activation, potentially optimizing neuromuscular function and offering a beneficial approach in the rehabilitation of knee injuries such as ACL ruptures and patellofemoral pain syndrome. Future research should focus on investigating the long-term effects and explore various manipulation techniques to further clarify their impacts on muscle activation and rehabilitation outcomes.

## Figures and Tables

**Table 1:** Demographic and anthropometric information.

	Experimental Group (n = 15)	Control Group (n = 15)
Men (n)	11	11
Women (n)	4	4
Age (years)	21.9 ± 1.7	22.1 ± 1.8
Height (cm)	178.3 ± 9.7	181.5 ± 9.5
Body mass (kg)	74.4 ± 11.6	76.6 ± 16.2
Body mass index (kg/m^2^)	20.8	21.1
Knee extension (Nm)	218.7	215
Fat tissue (%)	15.9	15.6

**Table 2:** Descriptive statistics (mean ± standard deviation) and inferential statistics comparing within-group differences (post–pre) for the main outcomes.

Outcomes	Experimental Group (n = 15)	Control Group (n = 15)
VL mean amplitude of EMG_RMS_ (µV)		
Pre	372.5 ± 211.8	414.1 ± 235.5
Post	397.5 ± 216.7	381.4 ± 215.4
Within-group difference (*p-value*|*d*)	*p* = 0.125|d = 0.117, trivial ES	*p* = 0.048 *|d = −0.145, trivial ES
VM mean amplitude of EMG_RMS_ (µV)		
Pre	377.4 ± 191.6	521.3 ± 267.5
Post	473.0 ± 175.7	414.3 ± 211.6
Within-group difference (*p-value*|*d*)	*p* < 0.001 *|d = 0.521, small ES	*p* < 0.001 *|d = −0.447, small ES
RF mean amplitude of EMG_RMS_ (µV)		
Pre	264.7 ± 103.5	340.4 ± 142.3
Post	292.2 ± 89.6	313.7 ± 131.9
Within-group difference (*p-value*|*d*)	*p* = 0.086|d = 0.285, small ES	*p* = 0.096|d = −0.195, trivial ES
VL max amplitude of EMG_RMS_ (µV)		
Pre	526.2 ± 268.5	542.2 ± 314.0
Post	526.3 ± 294.5	497.9 ± 275.1
Within-group difference (*p-value*|*d*)	*p* = 0.995|d < 0.001, trivial ES	*p* = 0.193|d = −0.150, trivial ES
VM max amplitude of EMG_RMS_ (µV)		
Pre	600.2 ± 315.1	737.3 ± 491.4
Post	688.3 ± 308.6	608.3 ± 388.8
Within-group difference (*p-value*|*d*)	*p* = 0.019 *|d = 0.283, small ES	*p* = 0.001 *|d = −0.293, small ES
RF max amplitude of EMG_RMS_ (µV)		
Pre	341.7 ± 122.9	436.5 ± 180.7
Post	374.9 ± 115.8	400.0 ± 166.0
Within-group difference (*p-value*|*d*)	*p* = 0.101|d = 0.278, small ES	*p* = 0.072|d = −0.211, small ES
VL median frequency of raw SEMG signal power spectrum (EMG_MED_, Hz)		
Pre	51.9 ± 7.2	55.5 ± 7.7
Post	55.2 ± 4.9	56.5 ± 7.2
Within-group difference (*p-value*|*d*)	*p* = 0.017 *|d = 0.545, small ES	*p* = 0.458|d = 0.134, trivial ES
VM median frequency of raw SEMG signal power spectrum (EMG_MED_, Hz)		
Pre	53.9 ± 11.3	55.4 ± 6.7
Post	55.3 ± 6.1	54.1 ± 6.1
Within-group difference (*p-value*|*d*)	*p* = 0.484|d = 0.161, small ES	*p* = 0.495|d = −0.203, small ES
RL median frequency of raw SEMG signal power spectrum (EMG_MED_, Hz)		
Pre	76.2 ± 16.0	77.3 ± 14.7
Post	75.3 ± 17.2	79.9 ± 13.8
Within-group difference (*p-value*|*d*)	*p* = 0.592|d = −0.054, trivial ES	*p* = 0.127|d = 0.182, trivial ES

ES: Effect size; *: significantly different at *p* < 0.05; rectus femoris (RF), vastus lateralis (VL), and vastus medialis (VM); surface electromyography (SEMG); median frequency (EMG_MED_); Hertz (Hz); microvolt (µV).

**Table 3:** Descriptive statistics (mean ± standard deviation) and inferential statistics comparing within-group differences (post–pre) and between-group (experimental vs. control) for the vastus lateralis (VL)–vastus medialis (VM) ratio.

Outcomes	Experimental Group (n = 15)	Control Group (n = 15)
VL/VM ratio—mean amplitude of EMG_RMS_ (µV)		
Pre	1.18 ± 0.73	0.85 ± 0.36
Post	0.83 ± 0.28	0.98 ± 0.40
Within-group difference (*p-value*|*d*)	*p* = 0.007|d = −0.693, moderate ES	*p* = 0.878|d = 0.342, small ES
VL/VM ratio—max amplitude of EMG_RMS_ (µV)		
Pre	1.16 ± 0.99	0.83 ± 0.37
Post	0.83 ± 0.28	0.91 ± 0.39
Within-group difference (*p-value*|*d*)	*p* = 0.044|d = −0.520, small ES	*p* = 0.412|d = 0.211, small ES
VL/VM ratio—median frequency of raw SEMG signal power spectrum (EMG_MED_, Hz)		
Pre	1.09 ± 0.37	1.09 ± 0.37
Post	1.07 ± 0.15	1.08 ± 0.42
Within-group difference (*p-value*|*d*)	*p* = 0.674|d = −0.077, trivial ES	*p* = 0.833|d = −0.025, trivial ES

Vastus lateralis (VL) and vastus medialis (VM).

## Data Availability

The data can be provided upon a reasonable request to the corresponding author.
